# Implementation of Casemix system in Hospital Universiti Sains Malaysia by using UNU-CBG grouper: is it feasible to support apex programme?

**DOI:** 10.1186/1472-6963-12-S1-I5

**Published:** 2012-11-21

**Authors:** Rosminah Mohamad

**Affiliations:** 1 School of Health Sciences, Universiti Sains Malaysia, Kubang Kerian, Kelantan, Malaysia

## Background

The framework of “Accelerated Programme for Excellence” (APEX), modeled on the German Universities Excellence Initiative, is profoundly important and decisive initiative for Malaysian higher education. The selection of USM under APEX initiative is based on the university's state of readiness, preparedness and transformation plan for change that focused on diagnostics, medical biotechnology, waste management, pharmaceuticals, nano-technology, membrane technology and vaccines. Becoming an APEX University, HUSM indeed is committed to achieve certain goals in their functions as teaching and referral hospital in the east cost of Peninsular Malaysia. One of the priority goals is to ensure that resources and allocation for each department within hospital is sufficiently distributed to ensure health care delivery is efficient and maintain at high quality to the patient. Selection of UNU-CBG Grouper is justified from the universal and dynamic functions of this Grouper that able to cater for various severity levels and able to capture an Acute (in-patient and outpatient), Sub-Acute (moderately complex cases) as well as Chronic Case (long stay cases) patients.

## Study objective

To preliminary observe the feasibility of using UNU-CBG Grouper as a grouping tool for Casemix HUSM.

## Methodology

Data mining of inpatient record discharged from HUSM within fiscal year 2009 and 2010 was done from Hospital Information Management System (HIMS). Data was transformed into Excel formatted sheet and exported into UNU-CBG Grouper software for further categorization and grouped into MY-DRGs and CBGs based on UNU-CBGs code classification system. Since HUSM is yet having the hospital tariff, UKMMC tariff was applied in this study by taken into consideration various similarities identified between these two teaching hospitals. We assumed the hospital tariff more or less will be the same between HUSM and UKMMC for us to fit the purpose of this preliminary study. MY-DRG codes for HUSM were generated from the Grouper, whereby the CBG cost weight was generated via integration of CCM software. Therefore, the CBG cost for HUSM patients was then determined. In order to observe the variance of charges between patient charges charged based on the Payment Guideline Schedule set by Finance Unit, HUSM and CBG cost, patient charges for three departments; Surgery, Orthopedics and Obstetrics, and Gynecology departments was reviewed. The charges were then quantitatively analyzed for the charges differential.

## Result

HUSM respectively exhibit 747-bed complement and occupancy rate of 68 patients per bed. The hospital has 5 days ALOS. Department of Surgery (4,932 admission), Orthopedics (3,694 admission) and, Obstetrics and Gynecology (8,525 admission) are among top ten departments that received highest admission within this two years. 83.6% (15,187 cases) of the admission were cases from A & E Department. The ALOS for each department in this study was 4 days, 6 days and 2 days, respectively. Average charge on patients that based on itemized procedures and consumables compared to CBG costs showed relatively low charges and significantly different at p < 0.005 with the mean different of RM5, 356.92 (SD- RM3,409.48; SE- RM695.96).

## Discussion

The importance of transforming HUSM to a more efficient hospital management system clearly presented from this pilot study. This scenario may contribute to the lower efficiency and low quality of healthcare services due to the insufficient budget allocated each year that is traditionally based on the previous year hospital expenditure. Unlike the traditional per diem costing (which is very crude) costs that daily rates are established for specific hospital departments and represent the average cost of hospitalization in specific departments, costs per weighted case capture the cost of hospitalization of a patient in a specific condition and are usually classified according to clinical diagnoses. Interestingly, the DRG system allows only one DRG assignment per patient episode, whereby payment will includes all services that occur between hospital admission and discharge. Grouping patients in this manner allows hospitals to evaluate and manage costs by DRG or groups of DRGs. Hope this research could deliver beneficial information to health care provider in HUSM and provide Health Campus community with such an up-to-date hospital management system.

## Conclusion

Minimal refinement on the coding and the framework on the HIMS is needed in order to cater the requirement for the Casemix System in HUSM. Decision to implement Casemix System as a hospital management tool is possible to enhance efficiency and quality of hospital delivery system in HUSM. Hence, aligned with the mission to support the APEX programme in USM. Therefore, it will be taken with high expectation to achieve efficiency, solidarity (fairness and equity).

